# The dynamic nature of leader–member exchange relationships in health-care organizations

**DOI:** 10.1108/LHS-06-2022-0073

**Published:** 2022-12-23

**Authors:** Sari Hirvi, Sanna Laulainen, Kristiina Junttila, Johanna Lammintakanen

**Affiliations:** Department of Health and Social Management, University of Eastern Finland, Kuopio, Finland; Nursing Research Center, HUS Helsinki University Hospital, Helsinki, Finland; Department of Health and Social Management, University of Eastern Finland, Kuopio, Finland

**Keywords:** Leadership, Nursing, Role, Dynamic, Participation

## Abstract

**Purpose:**

This study aims to make visible the dynamic nature of leader–member exchange (LMX) in the changing realm of health-care leadership.

**Design/methodology/approach:**

The qualitative study used an open questionnaire, which was distributed amongst nursing staff and managers at a Finnish public university hospital.

**Findings:**

The participants described partly LMX theory, but the leader-member relationship was also influenced by the organizational culture and the existing management practices. Nursing staff were found to have a more variable and dynamic role in the LMX relationship than has previously been reported. The research therefore provided novel information for the field of health-care research.

**Research limitations/implications:**

The presented research was limited by the content of the data, as the collected single narratives were rather short; however, the fact that a large number of narratives were collected from diverse participants strengthened the ability to reliably answer the research questions.

**Practical implications:**

Although the participants described partly LMX theory, the leader–member relationship is also influenced by the organizational culture and existing management practices; the finding that nurses have more variable roles in LMX relationships in the health-care context was new insight in this field. Therefore, the presented findings can help decision-makers change the current, perhaps antiquated, leadership practices at health-care organizations.

**Originality/value:**

This study provides new insight into the field of LMX research in terms of the important role of nursing staff, the organizational factors that influence the LMX relationship and the dynamic nature of LMX relationships.

## Introduction

The retention of skilled nursing staff is one of the most important issues facing modern health care, many health-care organizations need to improve leadership and the related organizational factors to provide a positive working environment for all health-care professionals. Previous studies (VanOyen, 2005) have shown that decentralized organizational structures and nursing staff members’ perceptions of the possibility to influence their work are important to the retention of staff. Moreover, staff participation and autonomy have been linked with job satisfaction (Schriesheim *et al.*, 1998), high-quality patient outcomes (Weston, 2008), nursing staff commitment and high overall well-being (Thornton Bacon *et al.*, 2019). In modern leadership, the autonomy of staff, joint decision-making and collectivity have emerged as central themes (McKee *et al.*, 2013), whereas awareness that leadership is a relational process has grown (Fairhurst and Uhl-Bien, 2012). This shift in leadership toward collective and participative activities is also visible in traditional hierarchical organizations, such as hospital environments (Joseph-Richard and McCray, 2022; De Brún and McAuliffe, 2020). In the context of leadership, collectivity often refers to various shared, relational and decentralized leadership practices (Eckert *et al.*, 2022), which are often related to the ability of staff to participate in decision-making and various work groups focusing on the development of patient care (Katrinli *et al.*, 2008). Collectivity in an organization also refers to the extent that the expertise of nursing staff is utilized in general management practices (Pursio *et al.*, 2021).

Leader–member exchange (LMX) theory is often used in leadership development and to study the relationships between a leader and team members. According to the traditional approach (Graen and Uhl-Bien, 1995), members predominantly participate by performing assigned formal tasks, and only in the most advanced phase of the LMX relationship are members allowed greater freedom to make work-related decisions and suggestions. Previous studies have confirmed that the consideration of staff concerns and enabling staff participation are important factors for the development and maintenance of high-quality LMX (Choy *et al.*, 2016; Katrinli *et al.*, 2008). Recent studies have found new elements to be connected to the LMX relationship, namely, the importance of individual values and organizational culture (Terpstra-Tong *et al.*, 2020), along with the connection between LMX relationships and team performance (Seo *et al.*, 2018). Moreover, Irshad *et al.* (2022) recently reported that increasing the autonomy of nursing staff can promote LMX relationships. In contrast, authoritarian leadership and power distances have been found to weaken LMX relationships (Siddique *et al.*, 2020).

LMX research has increasingly shifted attention to the member’s role in the mutual relationship, yet the field lacks studies that describe the member’s role, along with the associated factors, in depth. Nursing staff participation and autonomy have been studied in the past but research has focused – rather unilaterally – on nurses’ autonomy in clinical patient work and the associated decision-making (Thornton Bacon *et al.*, 2019). In this way, there is limited knowledge about the leadership perspective in the context of nursing. Moreover, there is a lack of research into how the role(s) of nursing staff have changed during management and organizational reform. We address this research gap by investigating the member’s role in LMX relationships in the context of health-care organization.

### Theoretical background

LMX theory has its roots in Vertical Dyad Linkage (VDL) theory, which states that a leader creates an individual relationship with each team member (van Breukelen *et al.*, 2006). According to VDL, each member’s role, as well as the relationship to their superior, develops through a formal exchange process (Dansereau *et al.*, 1975). The role of a participant became a key issue early in the development of LMX theory and remains an important part of how an LMX relationship is constructed (Day and Miscenko, 2016). LMX development can be understood through the role-making model, which comprises three phases: role taking; role making; and role routinization (Graen and Scandura, 1987). Furthermore, an LMX relationship that has progressed through the three-phase continuum of role-making can be considered high-quality and characterized by partnership and informality (Graen and Uhl-Bien, 1995). Although the role perspective has always been a strong part of LMX theory, other perspectives have emerged as the theory has evolved; an example is the relationship-based approach, which recognizes that the development of a relationship is not only based on formal, task-based processes, but should also take into account other aspects, such as personal characteristics and factors related to positive treatment (Erdogan and Bauer, 2016). It is important to state that LMX development involves multidimensional factors such as interpersonal emotional expectations of each other’s (Hirvi *et al.*, 2021).

Even though LMX theory has evolved from a formal approach to a more multidimensional concept over the past few decades (Liden and Maslyn, 1998), little attention is still paid to the role of members and opportunities for participation. Instead, previous research has primarily focused on employee performance in relation to the quality of the LMX relationship (Park *et al.*, 2022). It is obvious that the modern organizational reality, which involves decentralized and participative practices, allows employees to assume different roles. A member’s traditional, role-oriented behavior may not contribute to the development of the LMX relationship; however, improving members’ opportunities to negotiate their work-related responsibilities and roles can positively influence the quality of the LMX relationship (Wang *et al.*, 2022). The focus of LMX research is shifting towards a more collective (Song *et al.*, 2018) and employee-centered approach (Afzal and Rasid, 2021), which reflects that employees have a more active role in the LMX relationship than previously described. This shift towards collective leadership in dyadic relationships may not be easy. This is because the success of collective leadership in health care does not only depend on the development of relationships between members and the leader but is also influenced by tensions at the organizational level, which are reflected in the dyadic relationship between the leader and the follower. Currently, this dynamic has not been considered strongly enough in the literature base (Gibeau *et al.*, 2020). In this study, we describe the member’s role in the LMX relationship by taking into account recent recommendations that LMX relationships should be studied using a broad perspective that takes into account various roles (Scandura and Meuser, 2022). Furthermore, we have paid attention to recommendations that LMX quality should not be studied by defining managers' and members' experiences based on a strong theoretical construction, but rather studied comprehensively in the context in which the relationship occurs while considering that the reciprocity and nature of the relationship is constantly changing (Wilson and Cunliffe, 2022).

## Aim

The aim is to make visible the dynamic nature of the LMX relationship in the context of changing health-care organization. The present study was guided by the following research questions:

RQ1. What role(s) do nursing staff play in the construction of an LMX relationship?

RQ2. What kind of LMX relationship(s) are these participation roles associated with?

## Methods

### Participants and data collection

The data were collected from one university hospital in Finland which is committed to developing nursing staff participation and leadership based on the Magnet Recognition Program (ANCC, 2014). The open invitation was addressed to all of the units in the target organization that were known to develop their practices based on the Magnet Hospital model, as well as four other units that did not have clear indicators of staff participation and leadership development. Respondents were nursing staff and nursing leaders from different specialties and different wards (inpatient wards, intensive care units, outpatient wards and the operating ward). The data were collected using an electronic, open-ended questionnaire; the questionnaire was sent to a chief nursing officer, who was expected to distribute it across various units via an email invitation to nursing leaders, registered nurses and practical nurses. A respondent could decide to participate in the study by clicking on a link that leads to the questionnaire over a secure internet connection (secure socket layer). The questionnaire included six open-ended questions that touched upon themes of participative leadership that are highlighted in the Magnet hospital model, e.g. decision-making, autonomy and participation. We received a total of 128 responses, with 22 responses from nursing leaders and 106 responses from clinical nursing staff. The work experience of both the nursing staff and nursing leaders ranged from 3 months to 40 years. The nursing staff had an average work experience of 19 years, whereas the corresponding figure for nursing leaders in supervisory work was 15 years. Regarding background information, only the respondents’ ages and experience in the field were collected to guarantee that a diverse sample of leaders and nursing staff was obtained. The material was not further quantified, but was analyzed as textual material. The responses resulted in 44 pages of text (Arial font, size 12, line spacing of 1), with nursing staff responses contributing to 35 pages and nursing leader responses contributing to nine pages.

The employed questionnaire is as follows:

- How is the participation of nursing staff related to nursing practices, organizational development, and a unit’s activities?
- What the decentralised decision-making is in nursing and in the activities of your unit? What kind of opportunities it offers?
- Please describe independence in nursing, as well as what kinds of opportunities it offers to you and/or your team members?
- In which areas of the organization do nursing staff have the opportunity to participate in decision-making, and which factors promote or hinder this opportunity?
- Do you have anything else to add about opportunities for participation or decision-making amongst nursing staff?

### Analysis

The research was carried out in accordance with the qualitative research guidelines (Leavy, 2014, 1–5) and applied an interpretive approach (Thorne, 2014, pp. 108-111) in which the data were analyzed through membership categorization analysis (MCA); this method includes the assumption that people themselves produce descriptions and categories (Hester and Eglin,1997). Furthermore, the research was in line with the representative nature of membership categories, i.e. an individual’s speech is considered a conscious choice, and the researcher does not influence the categories in any other way than identifying and recording them (Sacks, 1995, pp. 40–56). Moreover, we used the concept of institutional order based on MCA (Psathas, 1999) to study the narratives linked to nursing staff participation by noticing cultural and structural elements of an organization. We use the term institutional order when we talk about the prevailing leadership culture and related practices in a hospital organization. Traditional institutional order describes the historically prevailing operating culture, i.e. specific professions define the boundaries of activity and decision-making, based on the old organizational and leadership culture (McKee *et al.*, 2013), which is characterized by practices based on traditional professional roles and generally accepted behaviors (Porter-ÒGrady, 2001), for example, *a doctor prescribes, a manager decides and a nurse cares*. The modern institutional order, constitute a culture in which knowledge, expertise and collectivity have high value (Hirvi *et al.*, 2021), and in which leadership is implemented in a modern and a multidimensional way (McKee *et al.*, 2013). An example of a modern leadership structure is the Magnet hospital model, which tends to embody the modern and independent position of nurses, by allowing the nursing staff as active and equal position in the organization’s multidisciplinary decision-making (Clure *et al.*, 2002, pp. 1-24).

**Figure 1.**
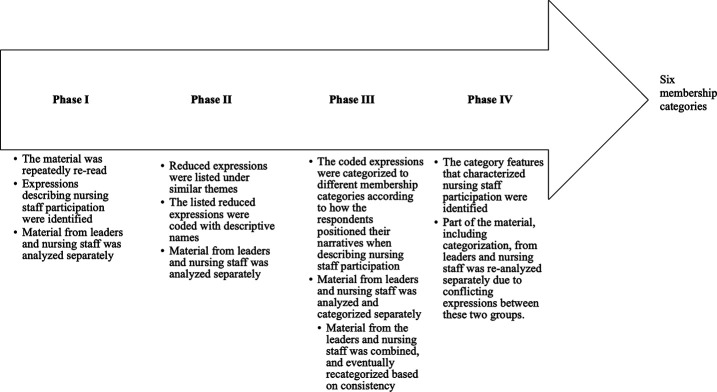
Analytical process

During the data analysis phase, the analytical unit was not a single respondent, rather, the text material was processed as a whole. Although the respondents’ answers were short, one respondent might describe different roles in his/her answers; in this way, a single respondent could not be considered as only representing a certain group or role. We paid attention to linguistic (written) expressions and how the informants described nursing staff participation. Moreover, we took advantage of the established principle of MCA that all of the analyzed actors (such as people, institutions and cultures) categorize each other into different categories (Hester and Eglin, 1997). We paid special attention to expressions describing fragmented nursing staff participation. The historical culture of hospitals, i.e. each profession includes traditionally defined roles, was taken into account when going through the data. Therefore, we paid attention to the linguistic means through which participants described factors related to the historical institutional order and potential fragmentation. The analysis included multiple phases, which are illustrated in Figure 1.

We began by identifying different expressions that respondents used when writing about the participation of nursing staff in nursing development and decision-making. We looked for expressions that seem to describe nursing staff participation (or its fragmentation), for example, “I can influence small decisions”. We listed the reduced expressions by theme and performed the initial coding by grouping similar reduced expressions according to how respondents described nursing staff participation and related events; for example, “bigger things coming from the top” or “we are carrying out a doctor’s orders” were listed under the same theme. Initially, we categorized data from the nursing staff and leaders separately, but we decided to combine the categorization due to a certain degree of similarity between the professions. During the categorization phase, we paid attention to subject and predicate pairs, such as “we are subordinate to doctors”, as well as extreme expressions and confrontation, for example, “everything is done for doctors”. We paid attention to the institutional order described above, and especially how the participants directly described this dynamic, e.g. “the nurse reports to the supervisor, who decides”. Although the narratives from leaders and nursing staff generally had the same tone, and we mainly analyzed the data together, we also considered the data from leaders and nursing staff separately to identify contradictory statements. This was done to ensure that differences in the narratives of leaders and nursing staff would be sufficiently taken into account. At the end of analytical process, we identified category features from the membership categories to develop a description of nursing staff participation.

## Results

### Membership categories describing the participation roles of nursing staff

**Figure 2.**
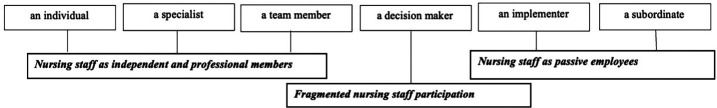
Membership categories and common features describing the participation roles of nursing staff

We identified six membership categories through which the respondents described nursing staff participation, namely, an individual, a specialist, a team member, a decision-maker, an implementer and a subordinate (Figure 2). In addition, we identified three common features of the narratives related to member categories that described the different roles of nursing staff as participants. The common features and membership categories of nursing staff are presented in more detail in the following text.

### Nursing staff as independent and professional members

The collected data revealed that nursing staff participation included an independent and professional role. Both leaders and nursing staff equally positioned nursing staff in the individual membership category and emphasized the value of nursing staff members’ personalities in relation to work assignments. Personality was a recurring attribute in responses from both leaders and nursing staff, with one respondent specifying “each own personality” and others stating that the norm or unspoken reality is “everyone is allowed to work independently, each “in their own personal way”. Individuality and freedom to express one’s own personality were described in a positive tone, and we did not identify any critical or contradictory expressions (fragmentation) from the respondents’ narratives. The opportunity for nursing staff to participate in decision-making and the freedom to act independently was expressed by describing competence, or by emphasizing the nurse's own work, as in one leader’s extract: “the nurse is the best expert in her own work”. A nurse’s competence provided them more freedom to participate and act independently based on their experience; as such, they were not considered as traditional subordinates to leaders. Considering the nursing staff as specialists disrupted the traditional institutional order. Hence, instead of only being expected to follow given regulations or instructions, expertise affords nursing staff the right to act independently, as the following extract shows:

I can make several decisions about patient care without a doctor and I do not have to ask for “permission” for my activities, my competence is trusted. (nursing staff)

In many responses, nursing staff were positioned in the team member membership category; in this way, nursing staff participation develops through multidisciplinary teamwork. Narratives related to this membership category included a neutral tone; this means that nursing staff are a strong part of health-care teams, as both leaders and nursing staff voiced that nursing staff are a part of this workgroup. Furthermore, no contradictory descriptions (e.g. group member/outsider) related to this category were noted:

Participation means that she feels that she is a competent member of a work community. Participation is about developing our work as part of a team (nursing staff).

According to these membership categories, nursing staff participation reflects practices which are in line with the modern institutional order, e.g. individuality, competence and team membership. These characteristics form a description of nursing staff participation in which all members are independent and competent professionals. It should be noted that these situations, with few exceptions, were related to the clinical patient work of nursing staff. We did not identify contradictions or fragmentation within these membership categories based on the leaders’ and members’ responses.

### Fragmented nursing staff participation

Although nursing staff participation were most clearly highlighted in the decision-maker membership category, the role was manifested most commonly in the context of clinical patient work practices.

The narratives associated with this membership category were particularly contradictory and constructed an image that nursing staff participation was mainly in line with traditional culture and practices. However, we identified positive and neutral expressions that described nursing staff participation in line with the modern institutional order. The fragmentation of the traditional institutional order was supported by respondents’ narratives that described the role of nursing staff as independent decision-makers. Furthermore, the nurses felt that they have an equal status alongside leaders and doctors. This is emphasized in the next extract, in which a member described themselves as an active decision-maker, with no confrontations with doctors or the leader:

I can negotiate the day’s plans with the doctor, etc. The leader trusts nursing staff and delegates tasks according to areas of responsibility (nursing staff).

Although we found evidence of nursing staff participation that agreed with the modern institutional order, we identified several linguistically colorful and strong expressions that indicated that certain aspects of nursing staff participation are linked to the traditional institutional order, i.e. historical professional roles. We identified respondents’ aspirations of what nursing staff participation should be like in the organization. These hopes were usually expressed alongside conflicting descriptions of reality. For example, nursing staff repeatedly placed the doctors in a decision-maker position, while situating themselves in the traditional role:

The nursing perspective should be more involved in decision-making at the higher level of the organization. At the moment, we are subordinate to doctors (nursing staff).

We identified fragmentation in nursing staff participation which manifested as perceived participation. Perceived participation was associated with narratives in which the nursing staff felt as if they were in the position of a decision-maker, but – in reality – the decisions were made by others. In these narratives, the nursing staff positioned managers or administrative actors as the decision-makers instead of nursing staff, for example:

things are decided elsewhere. We sit together seemingly democratically to decide, but our work is clearly guided by top-down command (nursing staff).

The leaders’ narratives included several references to decision-making. In these narratives, the nursing staff were mainly unconsciously positioned as passive actors while the leaders positioned themselves as the actual decision-makers; one leader wrote: “I ask for their opinions before making decisions”. This expression revealed that certain actions at the hospital were based on the traditional institutional order, in which the leader decides.

We found significant differences in how leaders and nursing staff discussed nursing staff participation. The leaders’ narratives revealed that leaders felt as though they enabled nursing staff participation by sharing opportunities for participating in decision-making. At the same time, some nursing staff respondents felt that they have no real opportunities for participation because the decisions had already been made by either leaders or doctors. Some of the nursing staff narratives demonstrated strong feelings of frustration (expressed in a critical tone) at the limited opportunities for participation in decision-making, e.g. one nursing staff respondent positioned clinical actors in a position where they cannot make decisions, and emphasized the gap between decision-makers and clinical actors: “At the unit level, decisions are made by supervisors and doctors, not nursing staff.”

Situations in which nursing staff are given the opportunity to be heard and express their views, but the actual decision is made by the leader, act as a turning point for the prevailing institutional order in the sense that they include elements of the traditional institutional order, yet also encompass elements of modern leadership and modern institutional order. An example would be attempts to involve nursing staff in decision-making, even though these efforts do not yet reflect active nursing staff participation. In the following extract, a leader gives the nursing staff a passive, even artificial, opportunity to be heard during decision-making, when in reality the expression emphasizes the leader’s role as a decision-maker:

Issues are discussed together in open meetings and decisions are justified to the staff in such a way that the employees feel that they have been heard (leader).

There were contradictory statements from leaders and members regarding the decision-making practices aspect of nursing staff participation. Although we recognized efforts to follow the modern institutional order, i.e. nursing staff have an active role in decision-making, many narratives confirmed that nursing staff participation is a complex phenomenon. The narratives of some leaders demonstrated an understanding that they should take advantage of practices that promote staff participation, yet the implementation of these practices was lacking.

### Nursing staff as passive employees

The narratives of both leaders and nursing staff included certain expressions that described the subordinate position of nursing staff *vis-à-vis* doctors and leaders, as well as the generally invisible position of nursing staff. The fragmentation of nursing staff participation, and the presence of the traditional institutional order, was emphasized by statements which reinforced the nursing staff members’ role of implementer and the leader’s role of unilateral decision-maker. The implementer membership category, which was related to nursing staff having a role of completing tasks and taking various instructions, included several extreme expressions of this position (e.g. ivory tower or poured to the neck). Several narratives described nursing staff participation as acting as implementers of the regulations/instructions. In many narratives, the instructions for how to care for patients came from the doctor, while general rules and regulations came from the top (managers or clinical experts); thus, there were many descriptions of how a nurse’s role was carrying out and following given instructions, as one registered nurse wrote: “Nursing staff must be involved in implementing changes, even if they cannot participate in planning or decision-making”. The fragmentation of nursing staff participation, as well as the presence of the traditional institutional order, was reinforced by statements which clearly described the nursing staff as implementers and top management as unilateral decision-makers. In some narratives, leaders also positioned themselves in a subordinate role (with the nursing staff) in relation to top management. The following extract shows a respondent’s experience that nursing staff (even the unit leader) do not have the opportunity to influence decisions or instructions given from the top.

projects are developed in the ivory tower of management and clinical experts and poured onto the neck of leaders and nursing staff (leader)

The subordinate membership category reflects an unspoken norm about whose voice matters in the organization and its governance. Many of these norms reflect previous eras, and should be understood to reflect a part of an organization’s history; nevertheless, it is important to state that they still strongly influence organizational operations in the modern time period. Narratives related to historical norms reflected fragmentation and a barrier to the active participation of nursing staff. The data included several instances in which the active participation of nursing staff was fragmented by the prevailing organizational hierarchy, as in the following leader´s extract:

An old-fashioned hospital hierarchy can be a pretty effective barrier to nursing staff participation. It can be difficult and even scary to question things in a hierarchical work community/organization (leader).

Many narratives included features of the traditional institutional order, such as fragmentation in the active participation of nursing staff as well as decentralized management reality. The traditional culture was referred to in narratives through terms such as hierarchy and nurses’ experiences of being a small player or category-bound action, as demonstrated in the following extract:

The conversation continues as before in the hospital; the hierarchy determines who speaks and who is silent. No one really listens to what that nurse says because everyone knows better and the nurse is a subjective and small player (nursing staff).

The existence of traditional organization culture at the studied hospital was reinforced by descriptions of “possibilities for influence are still rigid and bureaucratic” (nursing staff). Many respondents wrote about the prevailing culture and nursing staff participation in a tone which emphasized the need for reforms of the organizational culture so that nursing staff can have an equally active role in the organization as leaders and doctors; one leader wrote: “the culture needs to change, it requires courage”. The most critical descriptions by the leaders and nursing staff were related to “the old management culture” and its manifestation in the present moment. The narratives of both leaders and nursing staff revealed expectations that the leadership and management culture would shift in a way that would be more modern and equal. We did not identify expressions from the respondents’ narratives that promoted the development of nursing staff participation through this membership category. In contrast, we identified palpable differences in the narratives of leaders and nursing staff related to actual nursing staff participation and its implementation. This fragmentation was highlighted by descriptions of the nursing staff as subordinates who have no real or independent opportunity to influence or participate; moreover, the traditional profession-based roles (doctor prescribes, manager decides, nurse cares) were emphasized in several narratives. The membership category associated with the hierarchical organizational culture confirmed the existence of the traditional institutional order at the studied hospital.

## Discussion

The role of nursing staff as independent and professional participants in the LMX relationship was evident, and especially notable in relation to clinical patient care and the related decision-making in line with previous findings (Thornton Bacon *et al.*, 2019); more specifically, the active and independent participation of nursing staff is easier to notice in clinical work, but nursing staff participation sharply declines when decision-making shifts away from clinical patient care. The interpretation of the collected data agreed with prior research in that nursing staff competence supports their autonomy and active role in an LMX relationship (Brower *et al.*, 2000). The data analysis identified a strong team member membership category, which supports group performance in relation to member participation and the LMX relationship (Liden *et al.*, 2006). The findings about the nursing staff as independent and professional participants fully describe the partnership phase of the LMX continuum, i.e. parties can be active, independent and equal partners (Graen and Uhl-Bien, 1995).

The member’s role as a participant received particularly contradictory and variable support; thus, staff participation might be more problematic than is commonly described in the LMX literature. Nursing staff were sometimes positioned as equal decision-makers with leaders and doctors, but in some cases as passive respondents to leaders’ expectations of performance, in line with the formal the LMX relationship (Graen and Uhl-Bien, 1995). The narratives related to decision-making revealed a gap to which little attention has been paid, how leaders and nursing staff perceive the possibilities for nursing staff participation. This is an important topic for further research because several narratives revealed contradictory experiences between leaders and members. Most of the leaders’ narratives indicated that leaders feel that nursing staff are afforded sufficient opportunities to be heard and involved in decision-making. In contrast, the nursing staff narratives suggested that these situations were mainly artificial, i.e. top managers or doctors, instead of the nursing staff, were positioned in the role of participants and decision makers. The strongest contradictions between leaders’ and members’ experiences were observed in the decision-making category.

The role of nursing staff as passive employees corresponds to a phase in LMX development (Graen and Scandura, 1987) during which the relationship is formal, undeveloped and the member assumes a receptive and passive position; this emphasizes the dominant role of a leader, top management and administrative actors, as found in previous studies (Kvist *et al.*, 2013). In addition to the formal position based on LMX development, the passive employee membership category sheds light on the existence of a historical and hierarchical hospital culture (Fasoli, 2010) in which nursing staff have a subordinate role and limited opportunities to participate. Although the literature generally associates member participation with delegating tasks to members (Liden *et al.*, 1997), or the possibility of having the members’ voices heard (Budd *et al.*, 2010), these descriptions of participation manifested more as members’ expectations than actual practices in the present study. This agrees with previous findings in that power and hierarchy might force a member into a formal and subordinate role and limit their opportunities to participate in general development and decision-making (Porter-ÒGrady, 2020). The studied organization is very large, we believe that the hospital’s size may contribute to the strong prevalence of hierarchical practices; previous studies (Grinyer and Yasai-Ardekani, 1981) have shown that organizational structure and bureaucracy are influenced by an organization’s size, i.e. large companies use bureaucracy to manage complex organizational realities.

This study found nursing staff participation to be a complex and dynamic phenomenon. The complexity manifested as a strong underlying hierarchical organizational structure, along with traditional leadership practices, which seem to significantly affect staff roles in the organization. Surprisingly, and contrary to LMX theory, we found that doctors and organization hierarchy exert a strong influence on nursing staff participation. We believe that this dynamic is largely based on traditional hospital culture, i.e. doctors have a dominant position, and there is a presence of top-down governance. The complexity was noticeable in situations when nursing staff (sometimes also leaders) perceived limited opportunities to influence their own role as participant; for example, other actors had positioned them (either consciously or unconsciously) in a certain role without question. The dynamic nature of the LMX relationship was particularly evident in the fact that nursing staff had unstable nursing roles, which varied from situation to situation and required nurses to constantly negotiate and evaluate their position in relation to events, actors and prevailing leadership practices. In terms of decision-making practices, nursing staff balanced different roles with leaders, doctors, and top managers, which confirmed that the construction of nursing staff participation roles does not follow an established process.

In response to our research questions, we found that nursing staff participation constructed through six membership categories, which sometimes identify an independent and professional role for nursing staff – in line with modern leadership and an advanced LMX relationship – but also include contradictory experiences of nurses as passive actors in line with traditional leadership. Active nursing staff participation was significantly limited by traditional hierarchical and bureaucratic leadership structures. The findings confirm previous suggestions that the traditions and history of an organization strongly impact institutional development (Lonati, 2020). Our findings partly agree with what has been reported in previous research (Fagin and Garelick, 2004) in that nursing staff have a subordinate role in their profession; as such, despite great investments in staff participation and modern management, the traditional and formal management culture might still dominate in health-care organizations. This study showed that the role of a member in the LMX relationship is influenced by many external factors, such as the historical and institutional structures associated with the organization and the management practices based on them. Other actors in the organization may also exert a significant influence on the member role; thus, nursing staff do not always have the opportunity to influence or choose their role. The results of this study provide strong evidence that the role of a member in the construction of the LMX relationship is more variable, complex and dynamic than what is described in the three-phase continuum of LMX theory.

### Limitations and future research

This study was affected by certain limitations. The first limitation is related to the content of the data; many responses were only a few sentences long, so the presented findings are based on the interpretation of short narratives. However, we collected a large number of responses from diverse participants; this strengthens the reliability of the research. We confirmed the results of our analysis by interpreting certain data samples and using researcher triangulation. The second limitation is related to the small amount of leader respondents. We did not intend to draw universally applicable generalizations from the data. Nevertheless, although the study included a limited amount of leader respondents, we believe that our interpretations and conclusions represent – to a sufficient degree – the reality at the studied hospital. Clear similarities in the leaders’ narratives support this. The third limitation was that participation in the study was open; this means that respondents were free to participate in the study based on their own experiences, which could affect the tone of the responses as well as influence who decides to participate.

While the LMX relationship has been extensively researched, many factors which influence the process have not been fully elaborated. We feel that – based on the presented results – the factors that explain the dynamic and variable nature of the LMX relationship should be further studied. We recommend continuing the role-oriented investigation of LMX relationships through qualitative approaches to gain a wider understanding of the continuously changing role of a member, along with complex contextual factors associated with the organizational reality. We believe that the results are transferable to different leadership contexts and confirm that leadership is changing, especially in the health-care context. In terms of practical implications, the presented results support that nursing staff should be actively considered when developing health-care leadership; more specifically, an emphasis should be placed on the independent and participative role of nursing staff. Finally, more effort should be placed on identifying the background factors, obvious or concealed, which affect management practices.

## Conclusions

Based on this study, the relationship between leader and member at a Finnish university hospital does not fully follow the three-step continuum of LMX theory, which proceeds phase by phase only as a mutual relationship between leader and member. Based on the analyzed data, the development of the LMX relationship is strongly influenced by people and structures outside of the relationship; this indicates that the relationship is variable and adaptive, and therefore, the LMX relationship should be considered as a dynamic that emphasizes collectivity and variability rather than a mutual relationship that emphasizes bilateralism. The traditional theory cannot sufficiently describe the modern and changing role of nursing staff in the LMX relationship, as the LMX relationship in modern health care seems to be much more dynamic, variable, and multi-faceted than previously stated. Accordingly, LMX relationships should be considered from a role perspective instead of through the lens of exchange theory. Despite serious attempts to reform a health-care management into a participative and decentralized practice, the real-life perceptions and expectations of nursing staff suggest that the traditional leadership culture may be so deeply ingrained in organizational structures that these attempts are incomplete. We stress that management reform, and the related changes in the roles of nursing staff, require a broad understanding of the reform and its purpose, along with an interdisciplinary discussion about decision-making boundaries (Pihlainen *et al.*, 2018).
